# Model-Based Distribution and Abundance of Three Delphinidae in the Mediterranean

**DOI:** 10.3390/ani10020260

**Published:** 2020-02-06

**Authors:** Grigorios Karamitros, Georgios A. Gkafas, Ioannis A. Giantsis, Petros Martsikalis, Menelaos Kavouras, Athanasios Exadactylos

**Affiliations:** 1 Hydrobiology–Ichthyology Laboratory, Department of Ichthyology and Aquatic Environment, University of Thessaly, Fytokou str, 38446 Volos, Hellas; gkafas@uth.gr (G.A.G.); martsikalis@uth.gr (P.M.); menelaoskavouras@gmail.com (M.K.); 2 Department of Animal Science, Faculty of Agricultural Sciences, University of Western Macedonia, 53100 Florina, Hellas; igiants@agro.auth.gr

**Keywords:** Density Surface Models, *Stenella coeruleoalba*, *Tursiops truncates*, *Delphinus delphis*, distance sampling, marine mammal abundance

## Abstract

**Simple Summary:**

The distribution and abundance of three Delphinidae species (striped dolphin, bottlenose dolphin, and common dolphin) were investigated in an extended area of the Mediterranean Sea. Data from nondesigned transect line surveys were modeled in order to investigate important marine areas for marine mammals. The results indicated that the environmental covariates significantly affecting marine mammals’ distribution/abundance were depth and distance from 200 m isobaths for striped dolphin, latitude/longitude and depth for bottlenose dolphin, and latitude/longitude and chlorophyll concentration for common dolphin. Maps of predictions were designed in order to communicate the results of important hot spot areas throughout the Mediterranean.

**Abstract:**

Monitoring of Delphinidae species population patterns in the Mediterranean Sea was carried out in a sequence of surveys employing different approaches. Data from seven-year surveys with small catamaran sailing boats were analyzed under model-based approaches. Density Surface Models were used to produce spatial distribution prediction of three Delphinidae species (*Stenella coeruleoalba, Tursiops truncatus*, and *Delphinus delphis*) in an extended study area covering much of the Mediterranean Sea. A classical distance sampling protocol was applied in order to calculate the detection probability of clusters. Static (depth, slope, distance from the coast, and distance from isobaths of 200 m) and nonstatic (sea surface temperature and chlorophyll) variables were used to predict the species distribution/abundance in a generalized additive model context. *Stenella coeruleoalba* was found to be the dominant species, with an extended distribution in the study area; its abundance was significantly affected by both depth and distance. *Tursiops truncatus* and *Delphinus delphis* illustrated a significant abundance correlation with depth and chlorophyll, respectively, while both species showed a robust longitude correlation. Our model pinpoints the significance of nondesigned transect line surveys, suggesting the importance of specific habitat areas for future monitoring and conservation aspects of marine mammals.

## 1. Introduction

The importance of marine mammal monitoring in the Mediterranean basin has been highlighted by a series of studies not only for evolutionary, adaptation, and subpopulation patterns of these species in time and space [1,2,3,4] but also in a framework of monitoring abundance and distribution in different strata and the factors that may affect such patterns [2,5,6,7,8,9,10,11,12,13]. 

Accommodating 7% of global biodiversity [14], the Mediterranean constitutes, at the same time, a stage of multiple human pressures to marine species habitats [11,15]. The Mediterranean has complex patterns of water circulation [16]. Environmental conditions such as sea surface temperature and chlorophyll concentration could affect directly or in more complex ways the distribution patterns and abundance of regular biota residents, such as 12 marine mammal species [17]. Striped dolphin (*Stenella coeruleoalba*), bottlenose dolphin (*Tursiops truncatus*), and common dolphin (*Delphinus delphis*) are three widely distributed Delphinidae species throughout the Mediterranean Sea [18]. Striped dolphins inhabit offshore waters from Gibraltar to the Aegean Sea and the Levant basin, the Ligurian Sea, the Gulf of Lions, and the waters between the Balearic Islands and the Iberian Peninsula. Especially, the Alboran Sea is reported to be a significant geographical region for species abundance [18]. In contrast, bottlenose dolphins are found in mostly coastal waters and have been reported in the waters of Albania, Algeria, Croatia, Cyprus, France, Gibraltar, Greece, Israel, Italy, Montenegro, Morocco, Slovenia, Spain, Tunisia, and Turkey [19]. Common dolphins have been recorded in both pelagic and coastal waters [20], with the Alboran Sea reported to be an important feeding and breeding ground. Common dolphin has faced a dramatic population reduction during the last decades [6,21], maintaining its population occurrence in coastal Algeria; around Sardinia and Corsica; in the southeastern Tyrrhenian Sea; in the Strait of Sicily and around Malta; in portions of the eastern Ionian Sea, the Gulf of Corinth, and the Aegean Sea; and off southern Israel [6,18,20]. 

Cetaceans are reported as a totemic, or umbrella species since they respond to most of the criteria defined within the Marine Strategy Framework Directive [22], reflecting changes in function of marine ecosystems. Furthermore, a series of legislations and agreements, as well as, the Agreement on the Conservation of Cetaceans of the Black Sea, Mediterranean Sea and contiguous Atlantic Area [23], underlie the priority of monitoring such species. Recently, the IUCN Joint SSC/WCPA Marine Mammal Protected Areas Task Force has developed a classification scheme for Important Marine Mammal Areas (IMMAs) that is modeled on the successful example of Bird Life International’s Important Bird and Biodiversity Areas (IBAs) classification scheme [24]. Twenty six areas are characterized as IMMAs in the Mediterranean region [15]. These areas are defined as discrete partitions of habitat, important to marine mammal species that have the potential to be delineated and managed for conservation. Thus, dedicated systematic surveys of some kind of friendly platform (ship or aircraft) are an irreplaceable way of collecting valuable and reliable data, regarding the abundance and distribution patterns of marine mammal species [25]. 

As data from systematically designed surveys with equal coverage of extensive areas such as the Mediterranean are hard to obtain, a series of studies has been conducted in order to provide novel, model-based approaches to investigate variables (dynamic and static) which affect the distribution of Delphinidae species [26,27,28,29,30,31,32,33]. These variables represent not only regions of different habitat types but also include a variety of oceanographic characteristics such as sea surface temperature and chlorophyll concentrations. Modeling techniques such as species distribution models (SDMs) have been developed in order to predict spatially distributed species [26,28,29,31] when evaluating the relationship between observations and environmental parameters. Models in this context provide knowledge of the ecological processes affecting the distribution of marine mammals and make it possible to predict species abundance by pinpointing the relative importance of specific habitats [29]. Moreover, Sillero (2011) [34] proposed that ecological niche model (ENM) is a better term than SDM, as it refers directly to the ecological niche theory (habitat vs. species distribution). ENMs can be applied and be useful for rare species, where detailed distribution data at sea are difficult to obtain [35]; variables such as species movement ranges, habitat preferences, and potential population breaks, in addition to ecological barriers, could be investigated [34].

Density Surface Models (DSMs) were developed in the Generalized Additive Model (GAM) framework in order to produce spatial maps of abundance prediction [32,36,37,38], as well as data from opportunistic random nonsystematically designed surveys, providing helpful directions to policy makers and stakeholders. In DSMs, line transect survey data are used to fit a detection function to obtain detection probabilities for clusters followed by a spatial part of the model which uses the GAM framework. GAMs are constructed with per-segment counts or estimated abundance as the response with either counts or segment areas, corrected for detectability [32].

In the present study, the abundance and distribution of striped dolphin (*Stenella coeruleoalba*), bottlenose dolphin (*Tursiops truncatus*), and common dolphin (*Delphinus delphis*) were modeled from a seven-year, nondesigned transect line dataset. Our model-based approach was that of DSM, performed in an extended study area of the Mediterranean in order firstly to test its “predictive” power and secondly to illustrate species’ biogeographical patterns. Differences in spatial distribution derived from the examination of the environmental characteristics as variables which affect the three Delphinidae species distribution and abundance could reflect differential species-specific strategies in habitat occupation or different tolerances to anthropogenic impacts. Conclusively, variables which significantly affect distribution patterns could be proposed as remarkable changes of potential population reductions, including the case of common dolphins in the Mediterranean basin [6].

## 2. Material and Methods

### 2.1. Study Area

**Figure 1:**
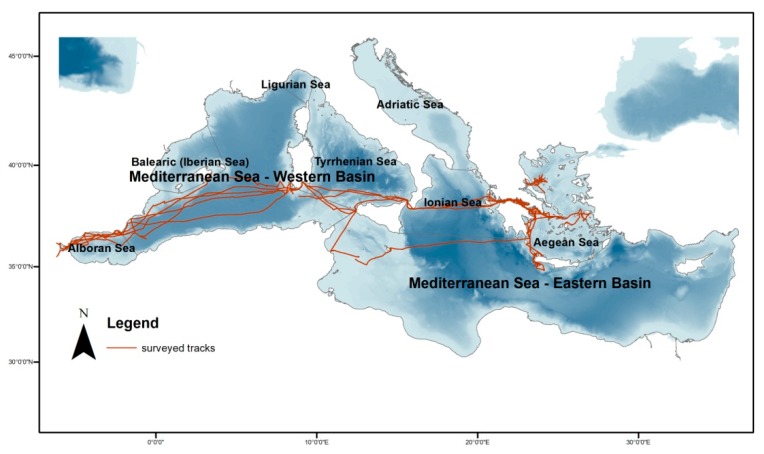
Surveyed region and studied subregions along with catamaran tracks.

The study area consisted of a significantly large part of the Mediterranean Sea (34°30’ N, 40°15’ N and 5°30’ W, 28°30’ E) including parts of the Alboran Sea, the southwestern basin, the south Tyrrhenian Sea, the Ionian Sea, and the Aegean Sea (Figure 1). Spatial limits of the study area were defined in order to avoid extrapolation to geographical regions beyond the boundaries where sampling originally occurred [39]. Surveys covered a variety of habitats such as oceanic areas (deeper than 2000 m), continental slope habitats (200–2000 m), and neritic coastal habitats (less than 200 m depth).

### 2.2. Data Collection

Data were collected with distance sampling techniques [36,40,41] by nonsystematically and systematically designed transect lines. Dedicated shipboard surveys were conducted from 2003 to 2007 with random transect lines and from 2018 to 2019 with designed zigzag, equally spaced transect lines with automated algorithms in the Distance 7.1 software (Figure 1) [36]. For all surveys, a 14 m catamaran boat with a diesel engine was used. The presence of cetaceans was assessed visually by observers scanning a 90° angle to the left and the right of the boat’s course and 3 m above the sea level. At the on-effort status, at least one observer was searching forward and to the side of the platform, minimizing the possibility of responsive movement prior to detection and recording the data.

The “passing” and “closing mode” approaches were applied depending on the weather conditions. At closing mode, the searching effort was stopped upon a sighting and started again when the sighting ended, with the aim of better species identification. GPS information (latitude/longitude variable) was recorded every 10 s, and sightings and environmental conditions were recorded in the Access Database using Logger 2000 and 2010 software (IWFA). A “sighting” was defined as a group of animals seen at the same time, showing similar behavioral characteristics. A laser range finder and 10 × 50 reticle binoculars with an internal compass were used for measurements of distance and angle, in order to calculate the perpendicular distance of the animal cluster from the course line [21]. Video and photographs were also captured for later analysis of groups, in order to investigate the species composition of the group and the number of individuals. For all on-effort statuses, the weather condition was up to 4 on the Beaufort scale, with good visibility, and the ship traveled at a low speed (6 kn) and stable heading. 

For environmental explanatory variables (Table 1), chlorophyll surface concentration (CHL; Mg m^−3^) and sea surface temperature (SST; °C) imagery from 8 days at a resolution of 4 km measurements was downloaded from NASA Ocean Color [42]. MODIS-SMI products for the study area were processed using the SeaWiFS Data Analysis System (SeaDAS) software and the GIS environment ArcMap 10.1 [43]. Covariates such as depth, slope gradient (hereafter slope), distance from coast, and distance from 200 m contour were computed using ArcMap 10.1 GIS software with EMODnet data [44]. All spatial data were transformed from WGS84 (geographical coordinate system) in Europe Lambert Conformal Conic Projection (projected coordinate system) in ArcGIS 10.1, in order to be manipulated in our analysis.

### 2.3. Data Analysis and Modeling Framework

Abundance estimations were made with the modeling of spatial patterns in animal density in the context of DSM, since this method does not require systematic or random sampling of the survey region with uniform coverage. DSM was used to predict the abundance of small cetacean clusters (striped, common, and bottlenose dolphins) [27,32] as a function of covariates that included SST; CHL; as well as topographic variables such sea floor depth, slope %, distance from coast, and distance from isobaths of 200 m.

DSMs model distance sampling line transect survey data using GAMs to identify the most important environmental variables explaining abundance patterns [27]. GAMs, like Generalized Linear Models (GLMs), use a link function to establish a relationship between the mean of the response variable and a “smoothed” function but can deal with highly nonlinear and nonmonotonic relationships between the response and the set of explanatory variables [45]. A two-stage methodological approach was applied for DSM according to Miller et al. (2013) [32]. Firstly, a detection function was fitted to the distance data. Half-normal and hazard rate detection functions were fitted to include observation-level covariates (sea states and animal clusters) that may have affected the detectability of clusters [19]. At the second stage, transects were divided into segments of approximately 1 km and the generalized additive model was fitted [45] to predict cluster abundances per transect segment, as a function of environmental and topographic covariates. A Horvitz–Thompson-like estimator was applied to estimate abundances in transect segments [46]. The goodness-of-fit statistics of each detection function was assessed with the Cramer–von-Mises and the Kolmogorov–Smirnov tests [19]. The best detection function was selected using the Akaike Information Criterion (AIC) [47]. All calculations were performed in R 3.6 [48], using the package “Distance” version 0.9.8 [49]. 

Data were analyzed with the multiple covariate distance sampling approach using the “Distance” [49] library of R 3.6 statistical language [48]. For the analysis, transect lines were split, in order to correspond to each environmental variable resolution, into contiguous segments of 1 km [32]. The expected abundance in each segment was modeled with Tweedie or a negative binomial distribution as a function of the covariates SST, CHL, depth, slope %, distance from coast, and distance from isobaths of 200 m [10]. The Tweedie distribution offers a flexible alternative to the quasi-Poisson and negative binomial distributions, as a response distribution when modeling count data [50]. To ensure that models were not overfitted, we removed variables that had values of *p* > 0.05 for bottlenose and common dolphins and values of *p* > 0.1 for striped dolphins in order to conclude more than one smoothing function, and then we refitted the models to ensure that all remaining variables had significant *p*-values [51,52].

We applied a variety of established metrics to compare the performance of the models built with the six different sets of predictors (SST, CHL, depth, slope %, distance from coast, and distance from isobaths of 200 m), including AIC, REML score, the percentage of explained deviance, and visual inspection of predicted and observed distributions during our cetacean surveys. Each of these models was then used to produce a final prediction on 4 km resolution grids responsive to a finer resolution of tested environmental factors.

## 3. Results

### 3.1. Three Main Dolphin Species Occurrence

**Figure 2:**
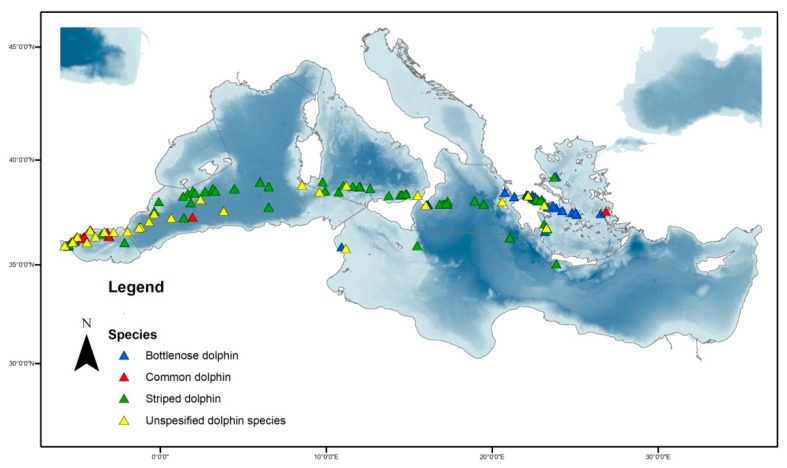
Distribution of visual mammal clusters observations. Each species is shown by a different color.

In a total of 5333 km of visual transects conducted under favorable environmental conditions, we recorded 137 small cetacean clusters (Figure 2) (68 of striped dolphin, 22 of common dolphin, 17 of bottlenose dolphin, and 30 of unidentified species). 

### 3.2. Detection Function

**Figure 3:**
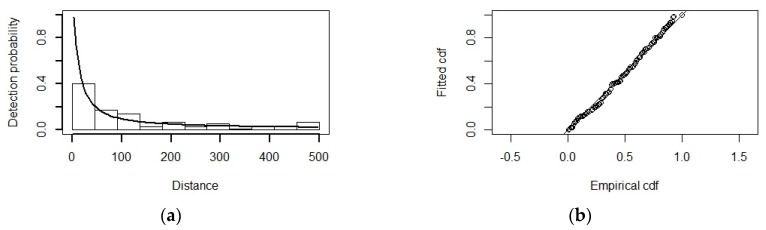
Plot of the fitted detection function (**a**) and goodness-of-fit plot (**b**) for the hazard rate model with sea state and cluster size as covariates.

To fit the detection function, all data were pooled in order to obtain robust estimates [41], whereas all three species share similar body size and sighting characteristics [53]. We selected a hazard rate key function (Figure 3a) with no adjustment terms with sea state (in Beaufort scale) and cluster size as covariates at the observation level by AIC. Observed distances were truncated at 500 m, based on the visual inspection of the histogram of distances [41], as well as comparing test statistics from the Cramer–von-Mises and Kolmogorov–Smirnov goodness-of-fit tests (Figure 3b). The average detection probability was 0.09, and the coefficient of variation was 0.26.

### 3.3. Density Surface Models and Predictions

#### 3.3.1. Striped Dolphin

**Figure 4:**
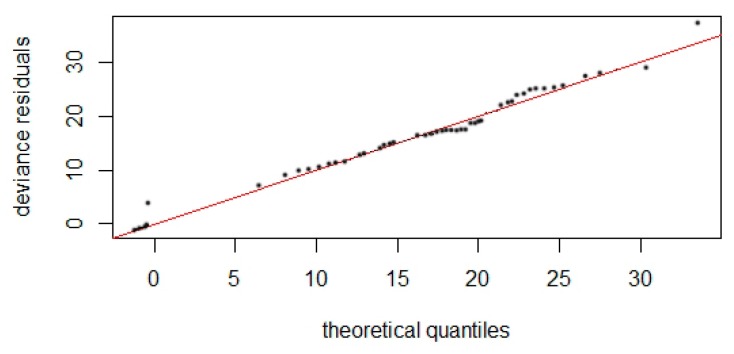
Quantile–quantile plot with Tweedie response distribution and the variables depth and distance from 200 m isobaths.

**Figure 5:**
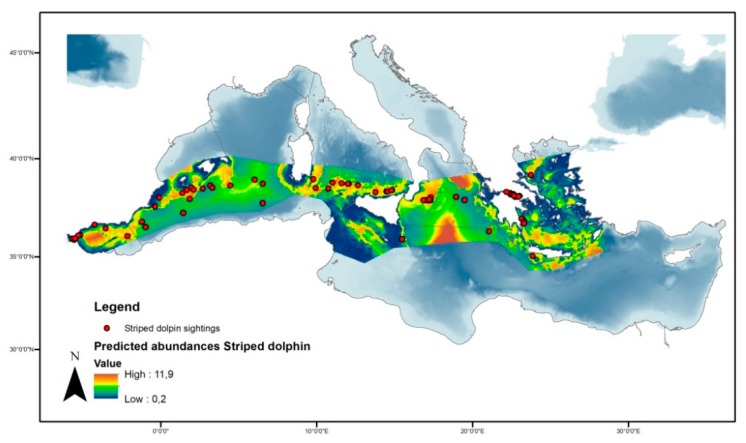
Spatial prediction of abundance for striped dolphins per cell in the studied area.

**Figure 6:**
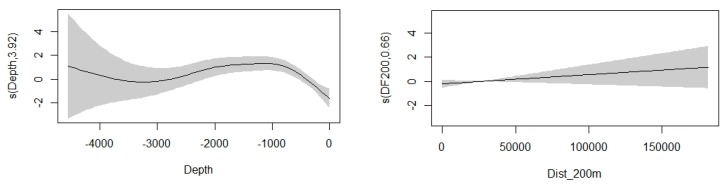
Smooth functions for depth and distance from 200 m isobaths. Grey shading corresponds to 95% confidence levels, and numbers in brackets on the vertical axis labels give the effective degrees of freedom of the term.

The density surface model with a Tweedie distribution and significant variables of bottom depth and distance from isobaths of 200 m provided the best fit for our data according to established criteria (Figure 4). The model predicted the total occurrence of 137.235 (95% CI = 72.638−259.280) individuals in the study area (Figure 5). The coefficient of variation from the GAM was 0.197 and the total coefficient of variation calculated with the delta method was 0.333. Deviance was explained at 10.1% for the model, with *p*-values of 6.54×10^−7^ and 0.088 for depth and distance from 200 m isobaths, respectively. Smooth functions for depth and distance from 200 m isobaths remained in the final fitted model (Figure 6).

#### 3.3.2. Bottlenose Dolphin

**Figure 7:**
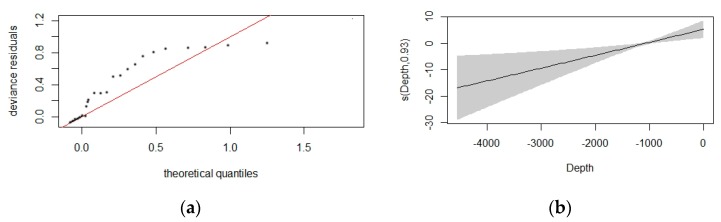
Quantile–quantile plot with negative binomial response distribution and the variables latitude, longitude, and depth (**a**). Smooth function is for depth (**b**). Grey shading corresponds to 95% confidence levels, and the numbers in brackets on the vertical axis labels give the effective degrees of freedom of the term.

**Figure 8:**
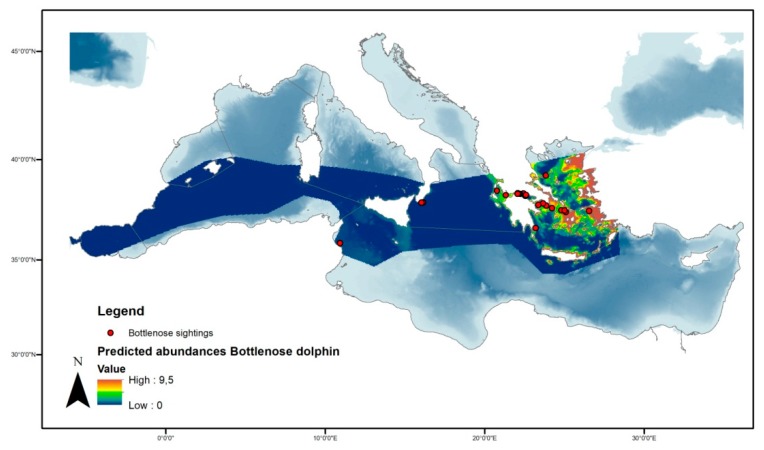
Spatial prediction of abundance for bottlenose dolphins per cell in the studied area.

The bottlenose dolphins from a series of models that best fit the density surface model with a negative binomial distribution and addressing significant variables of latitude, longitude, and depth are illustrated in Figure 7. The model predicted the occurrence of 11.353 (95% CI = 3.218−40.047) individuals in the study area (Figure 8) with the deviance explained at 53.8% and *p*-values of 0.0012 and 0.0008 for latitude/longitude and depth, respectively. The coefficient of variation from the GAM was 0.663 and the total coefficient of variation calculated with the delta method was 0.716.

#### 3.3.3. Common Dolphin

**Figure 9:**
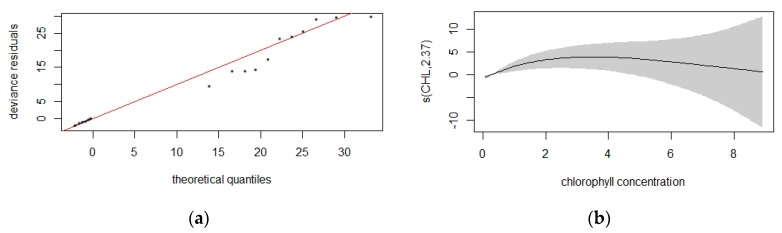
Quantile–quantile plot with Tweedie response distribution and the variables latitude, longitude, and chlorophyll (**a**). Smooth function for chlorophyll concentration (**b**). Grey shading corresponds to 95% confidence levels, and the numbers in brackets on the vertical axis labels give the effective degrees of freedom of the term.

**Figure 10:**
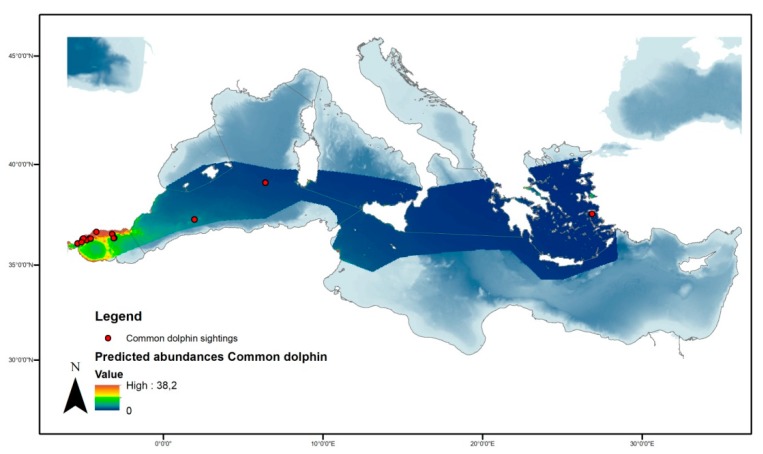
Spatial prediction of abundance for common dolphins per cell in the studied area.

According to our dataset for common dolphin, the density surface model with a Tweedie distribution and significant variables of latitude, longitude, and chlorophyll provided the best fit in comparison with the negative binomial (Figure 9). The model predicted the occurrence of 13.710 (95% CI = 58.68−32.033) individuals in the study area (Figure 10) with the deviance explained at 29.8% and *p*-values of 0.0067 and 0.0011 for latitude/longitude and CHL, respectively. The coefficient of variation from the GAM was 0.366 and the total coefficient of variation calculated with the delta method was 0.454.

## 4. Discussion

Models for spatial abundance prediction of marine mammals such as DSMs constitute a novel approach with a series of applications [27,32]. Spatial and temporal studies in the context of conservation, as well as biogeography of endangered marine mammals give extra tools, not only for a better understanding of the spatial and temporal deviance in abundance and density of the species [28,54,55,56,57] but also for monitoring these populations [31]. Furthermore, field survey data from such a wide area like the Mediterranean basin at spatial and temporal levels promote biogeographic approaches for low-density abundant species [55]. As distribution and abundance are influenced by a series of variables that are mostly difficult to obtain at a vast scale, DSMs can be characterized as comprehensively predictive rather than explanatory, suggesting the potential covariates that significantly affect spatial distribution [54]. However, spatial abundance and distribution predictions may propose the establishment of important areas for marine mammals (IMMAs) and environmental variables that affect distribution, helping in this way the protection of habitats from a series of activities such as marine traffic [56], military training, or seismic surveys. 

For the analysis of valuable cetacean survey data, a series of spatial statistical models has been developed, including spatially referenced covariates related with environmental predictor variables. A series of ecological niche models with a variety of approaches, such as species distribution, habitat distribution, or climatic envelope models, are different covariates for similar mechanistic or correlative models [34]. Particularly, SDMs develop empirical approaches correlated with field observations and environmental predictor variables, based on statistically or theoretically derived response factors [26,28]. Despite the challenges that SDMs face, modeling marine biota can be a sufficient method to obtain valuable information about phylogeographic patterns, especially in combination with genetic approaches [31]. Species habitat models also constitute spatial models which include habitat variables, allowing more reliable predictions of densities than traditional transect line analysis [29]. DSMs combine spatial modeling techniques involving not only environmental factors as explanatory variables but also distance sampling methodology, thus constructing a final model of two or more stages of abundance with a detection function to correct uncertainties [27].

The present study focused on a wide study area of high importance, namely, the Mediterranean basin, in order to combine sighting data from distance sampling with environmental or biogeographical variables. The limitations were related to uncertainty, such as temporal variability of environmental variables. Despite the fact that all environmental variables are according to the specific date of sampling for each transect, the optimum time frame could not be negligible. This temporal transition between different seasons may affect factors such as SST being statistically revealed as significant in our models. Bibliographically speaking, unavailable variables such as the occurrence of prey may affect the fine-scale species distribution [58,59]. Furthermore, one of the main limitations concerning the model-based analysis used is the risk of bias in abundance estimation due to model misspecification [27]. Regarding the distribution and abundance of rare species such as bottlenose and striped dolphins in our study, conclusions about the predictions need to be taken into account with caution due to a limited series of sightings. Nevertheless, our presented novel findings were subjected to fine, robust goodness-of-fit statistics and should be regarded as such, adding great value to the biogeography of marine mammals’ abundance in the Mediterranean.

The purely fitted models of the dataset took into account only significant explanatory variables in order to prevent overfitting [54]. However, despite the extended study area, due to reasons described by [54], our density surface maps showed that such models could capture important explanatory variables in distribution patterns. The striped dolphin was found to constitute the dominant species, with its consistent presence in most regions of the Mediterranean basin. Important factors that seem to favor its constant spatial distribution are depth and the distance from 200 m isobaths, a fact which is in accordance with similar previous studies [5,10]. This pattern was indeed seen elsewhere, where species behavior showed a local population structure, implying a differential ecology between inshore and offshore populations [2]. Moreover, it has also been suggested that striped dolphins prefer habitats deeper than 300 m [13,60], which was correctly verified from the significance of the depth variable in our model. 

Predictions of abundance regarding the common dolphin pinpoint the Alboran Sea as an important hot spot. Significant population abundance in the Alboran Sea seems to be related to an important feeding and breeding ground [6,61]. This fact could also be explained by the area’s high primary productivity, as indeed chlorophyll appeared to significantly influence our model. An established difference in the primary productivity of the eastern Mediterranean basin considered as oligotrophic, compared specifically with the western part in the Alboran Sea, is well documented [62]; this biogeographic pattern may also reflect recorded differences in feeding grounds of the common dolphin. 

The biogeography of marine mammals could be shaped by evolutionary or ecological forces and influenced by environmental instabilities, moreover by direct anthropogenic pressure. The three species in question in our study displayed clear-cut differences regarding the environmental variables that affect their distribution in large-scale modeling. Striped and bottlenose dolphins, for instance, have similarities in structural patterns revealing genetic distributions throughout their main boundaries in the Mediterranean [1]. With depth and distance from isobaths of 200 m being the main significant variables, the distribution of the striped dolphin displayed a wide population spread in almost every longitude of the studied area, in contrast to the bottlenose dolphin. Common dolphin, on the other hand, seems to be significantly influenced by chlorophyll concentrations. Moura et al. (2013) [58] recorded the significant relationship between primary productivity and common dolphin presence in their models, proposing the potential effect of prey. Therefore, these kinds of variations in environmental factors or physical characteristics that can affect the differential distribution between the three species may reveal divergent tolerances to changes in prey availability; the latter could be proposed as a likely case scenario for the decline of common dolphin populations in the central and eastern Mediterranean [1].

In a relevant larger spatial scale, it is worth mentioning that it was evidenced that bottlenose and common dolphin species had differential longitude preferences. Our findings backup the robust significant effect in spatial distribution of the static variables latitude and longitude for the bottlenose and common dolphins, while the variable depth seemed to be more informative for the striped and bottlenose dolphins. Since the Mediterranean Sea can be separated into western and eastern parts of the basin with well-documented divergent ecological characteristics [63], one could safely suggest divergent population dynamics strategies. Noteworthy is the fact that striped dolphins between the western and eastern parts of the Mediterranean display divergent genetic clusters, resulting from complex migration patterns and demographic scenarios, which may also be reflected by different ecological characteristics [2,4].

## 5. Conclusions

Among the dynamic variables SST and CHL, the CHL variable seemed to affect only the distribution of the common dolphin, a fact that may be interpreted from the documented higher primary productivity in the Alboran Sea [62]. Interestingly, the variable SST did not appear to significantly affect any of the Delphinidae species in our models. This fact, in our findings, may have been due to the small variation of SST in our study area corresponding to the habitat preferences of the species in question. Additionally, recorded differences in spatial distribution due to sea temperature variations may not reflect any habitat requirements but may be mostly due to different prey preferences [64]. According to our data, the bottom slope did not appear to correlate firmly with spatial distribution, which has been previously stated in the literature [20,65,66], a fact which is in contrast with depth. The latter may reveal that the habitat type is also related to disturbance from nearshore human activities [11]. The bottlenose dolphin seems to prefer mostly coastal habitats with depths around the isobaths of 100 m, a fact that is probably affected by their feeding strategies [11,67]. 

The present dataset that was acquired with the use of small catamarans adopting the basic principles of random distance sampling, despite the limited detection probability, may constitute an important key tool in monitoring marine mammal population divergent structures, confronting human pressure activities and climate change consequences. Data from surveys focused on Marine Protected Areas (MPAs) under sheer surveillance or ecotouristic approaches can provide, under certain conditions and circumstances, significant datasets for scientific analysis. Null hypotheses such as declining fish stocks, marine litter pollution, maritime traffic, direct killing, fishery bycatch, underwater noise pollution, and seismic surveys constitute and will continue to remain challenges which will lead, one way or another, to the evolution of adapted species and, at the same time, will enhance our efforts to monitor marine mammals in order to conserve natural resources.

Comparing species abundance can be challenging due to the fact that species communities often comprise many different aspects of abundance profiles, such as predation [68], social structure [69], kinship associations, demographic dynamics [4], and evolutionary forces. Further, different environments could drive different population structures in a widely distributed species in a given area [70].

## Figures and Tables

**Table 1:** Characteristics of the variables used in spatial analysis.

Variables	Name Used	Source	Spatial Resolution	Unit
**Depth**	Depth	GEBCO	1/16 arc	m
**Slope %**	Slope	GIS calculations	1/16 arc	Degree
**Distance from coast**	DFC	GIS calculations	-	m
**Distance from 200 m isobaths**	DF200	GIS calculations	-	m
**Sea surface temperature**	SST	MODIS (NASA (b), 2013)	4 km	°C
**Chlorophyll-a**	CHL	MODIS (NASA (b), 2013)	4 km	mg/m^3^
**Longitude/latitude**	x/y	Europe Lambert Conformal Conic	-	m
